# How Do Hunter-Gatherer Children Learn Subsistence Skills? A Meta-Ethnographic Review

**DOI:** 10.1007/s12110-017-9302-2

**Published:** 2017-10-09

**Authors:** Sheina Lew-Levy, Rachel Reckin, Noa Lavi, Jurgi Cristóbal-Azkarate, Kate Ellis-Davies

**Affiliations:** 1 Department of Psychology, University of Cambridge, Free School Lane, Cambridge, CB2 3RQ UK; 2 Division of Archeology, Department of Archaeology and Anthropology, University of Cambridge, Downing Street, Cambridge, CB2 3DZ UK; 3 Department Anthropology, University of Haifa, University of Haifa Mount Carmel, 31905 Haifa, Israel; 4 HEZI Aholkularitza Pedagoikoa, Iturriotz 23 3°A, 20500 Arrasate, Gipuzkoa, Spain; 5 Department of Psychology, Nottingham Trent University, Chaucer Street, Nottingham, NG1 4BU UK

**Keywords:** Learning, Forager, Life history, Meta-ethnography, Cultural transmission, Childhood

## Abstract

Hunting and gathering is, evolutionarily, the defining subsistence strategy of our species. Studying how children learn foraging skills can, therefore, provide us with key data to test theories about the evolution of human life history, cognition, and social behavior. Modern foragers, with their vast cultural and environmental diversity, have mostly been studied individually. However, cross-cultural studies allow us to extrapolate forager-wide trends in how, when, and from whom hunter-gatherer children learn their subsistence skills. We perform a meta-ethnography, which allows us to systematically extract, summarize, and compare both quantitative and qualitative literature. We found 58 publications focusing on learning subsistence skills. Learning begins early in infancy, when parents take children on foraging expeditions and give them toy versions of tools. In early and middle childhood, children transition into the multi-age playgroup, where they learn skills through play, observation, and participation. By the end of middle childhood, most children are proficient food collectors. However, it is not until adolescence that adults (not necessarily parents) begin directly teaching children complex skills such as hunting and complex tool manufacture. Adolescents seek to learn innovations from adults, but they themselves do not innovate. These findings support predictive models that find social learning should occur before individual learning. Furthermore, these results show that teaching does indeed exist in hunter-gatherer societies. And, finally, though children are competent foragers by late childhood, learning to extract more complex resources, such as hunting large game, takes a lifetime.

Humans have an exceptionally long pre-reproductive lifespan for our body size (Bogin 2006). Humans are also unique in our ability to transmit vast quantities of cultural knowledge from one generation to the next. This transmission of knowledge and accumulation of culture allows us to update our technologies and environmental knowledge in response to changing surroundings (Boyd et al. 2011; Laland 2004). Some have argued that this human emphasis on learning has shaped our especially long childhoods (e.g., Kaplan and Robson 2002). Since hunting and gathering has been humanity’s subsistence strategy for more than 90% of our evolutionary history, data from modern hunter-gatherer children can be and has been used to test theories about how knowledge transmission has shaped the evolution of our life history strategy (Marlowe 2005). And yet modern foragers are not direct analogues to the past, nor are they a homogenous group; it is their immense cultural diversity that makes the traits many foraging groups hold in common all the more striking. In addition, studying the social learning of foragers in particular can help us understand the diversity that exists among small-scale societies in general (Boyette and Hewlett 2017).

Unfortunately, of the few studies that exist on the topic of learning subsistence skills among foragers, only a handful employ a cross-cultural approach (e.g., Barry et al. 1957, 1959; MacDonald 2007). Yet sociocultural perspectives support the cross-cultural study of learning, in recognition of the interdependence of social and individual processes in the co-construction of knowledge (John-Steiner and Mahn 1996; Nielsen and Haun 2016). The few existing cross-cultural studies of hunter-gatherer learning usually focus on a particular skill, such as hunting, and thus fail to recognize how learning might be similar or different across various skill domains.

To address this gap, the present paper adopts a meta-ethnographic approach in order to understand how hunter-gatherer children from around the world learn subsistence skills. Our goal is to answer three main questions: first, how do hunter-gatherer children learn those subsistence skills necessary to survival? Second, how long does it take to learn those various skills? Finally, from whom do children learn subsistence skills? Our approach is novel; though other publications have used a systematic cross-cultural approach (e.g., Barry et al. 1957, 1959), the particular methods associated with a meta-ethnographic review have never been applied to these questions, though they are ideal for distilling patterns from broad data. By searching for learning behaviors in both quantitative and qualitative literature, a meta-ethnographic review process can help uncover trends that apply to foragers cross-culturally, as well as behaviors that stand out as culture-specific (Blurton Jones et al. 1994; Harkness and Super 1983). Findings can then, cautiously, be used to test theories about humanity’s foraging past. Before we describe our methods and results, we offer some background on human life history patterns, and outline features of social and individual learning in humans.

## Background

Primates in general, and chimpanzees, bonobos, and humans in particular, have adopted a long and slow life history strategy known as K-selection (MacArthur and Wilson 1967; Smith 1989). Like other K-selected species, we have relatively large bodies and invest heavily in a small number of offspring that take a long time to mature. Though humans are similar in size to chimpanzees, some of our life history traits do not fit the expected pattern for our clade. We have longer pre-reproductive lifespans, higher fertility, and shorter interbirth intervals than expected for our body size, even when considering the variability in human birth spacing and fertility (Chisholm 1993; Lancaster et al. 2000; Leigh 2001; Robson and Kaplan 2003). Primates have a period of infancy, from birth throughout the process of weaning. This is directly followed by juvenility, where individuals are independent from direct provisioning from parents but are not sexually mature. However, Bogin (2006) suggests that humans have inserted another life history stage between these: early childhood, defined as a period in which, though weaned, children still rely on adults for direct care (Bogin 1997).

Why do humans have this extended childhood? Kaplan and Robson (2002) argue that it is an adaptation for learning complex extractive subsistence skills, especially hunting. Kaplan et al. (2003) point to the fact that, during early childhood, children’s bodies grow relatively little, whereas their brains reach 95% of adult size by the time they transition into juvenility around age six (Bogin 1997, 2006; Konner 2010). Since humans make use of resources that require complex skill and knowledge to extract, investment in a large brain in early childhood sets the groundwork for complex learning later in life and thus increases future performance (Kaplan and Robson 2002; Kaplan et al. 2003). This investment in embodied capital, according to Kaplan et al. (2000), is a driving factor in the evolution of human ontogeny.

So by what mechanisms do children learn, no matter their subsistence context? Children can learn through play, participation, observation, and imitation. Play, specifically, is an important tool through which children learn community-wide social norms and practice their “chore curriculum” (Chick 2009; Elias and Berk 2002; Göncü et al. 2006; Lancy 1996, 2015). Play also serves as a key venue for developing skills such as harvesting and hunting (Bock 2002, 2005; Bock and Johnson 2004). Indeed, Bock and Johnson (2004) and Boyette (2016) found that children played less and worked more as they aged. More specifically, Bock (2002, 2005) and Bock and Johnson (2004) found that, as children grow older, play that emulated specific, complex adult activities, such as pounding grain or hunting, becomes less frequent, while actual participation in these activities increases. This suggests that play may provide children with an opportunity to practice complex activities. In addition, participating in adult activities alongside either adults or other children, such as gathering water or firewood, allows a child to develop the necessary competencies to complete these tasks independently (Gaskins 2000; Lancy 2012; Rogoff et al. 2003). Finally, in small-scale societies where adult activities are not segregated from those of children, children have ample opportunities to observe adults and to imitate their behaviors (Fouts et al. 2016; Gaskins and Paradise 2009; Odden and Rochat 2004).

Not only are children active imitators, they are also overimitators, defined as the imitation of a model’s relevant as well as irrelevant actions (Lyons et al. 2007), as demonstrated by various experiments in WEIRD—Western, Educated, Industrial, Rich, and Developed (Henrich et al. 2010)—societies. For example, in an experiment conducted by Lyons et al. (2007), a model demonstrated how to open a variety of containers through a series of relevant and irrelevant actions. The 3- to 5-year-old children involved in the study were asked to identify any irrelevant action after each demonstration. Though they did so successfully, when shown how to open the next container with relevant and irrelevant actions, children imitated the sequence modeled by the adult faithfully. Over and Carpenter (2012) argue that overimitation allows children to learn technologies and cultural practices whose meaning is opaque, allowing for fidelity of transmission across generations. On the other hand, children appear to be incredibly selective in how and from whom they learn (Meltzoff 1995; Over and Carpenter 2012, 2013). In one study, 14- to 18-month-olds imitated individuals who showed intentionality in their action, marked by the model saying “There!” If the same action seemed accidental—marked by the model saying “Whoops!”—children were less likely to copy the action (Carpenter et al. 1998). Some consider imitation and innovation the dual engines of cultural learning, as both are required for the evolution of cumulative culture (Legare and Harris 2016; Legare and Nielsen 2015).

Innovation, also known as individual learning, is especially adaptive when an environment is in flux, and when new, novel innovations must be generated to better adapt to ecological changes (Aoki et al. 2012; Boyd et al. 2011; Enquist et al. 2007). However, individual learning is costly, in that many attempts must be made before a useful innovation is developed (Boyd et al. 2011; Kline et al. 2013). Predictive models suggest that, in order to learn adaptively, social learning should occur early in life, and trial-and-error learning should occur later, once baseline competencies have been reached (Aoki et al. 2012). Successful innovative behaviors are then diffused throughout the social group.

Finally, children learn from a wide variety of individuals, including parents, other adults, and, importantly, other children. Vertical or parent-to-child transmission (Cavalli-Sforza et al. 1982; Hewlett et al. 2011) seems to be less conducive to innovation, meaning it is more common in stable environments where information need not change rapidly. Various studies have also noted that most vertical transmission is sex-segregated, meaning that mothers teach their daughters and men teach their sons (Chen et al. 1982; Hewlett and Cavalli-Sforza 1986). Oblique transmission takes place when other adults from the parents’ generation teach children. Oblique transmission is common for learning ceremonial practices, for example, where many members of a cultural group share the same information. Child oblique transmission is when older children teach younger ones. Horizontal transmission occurs within members of the same generation—in this case, children to children—and allows for the rapid diffusion of information. Thus, some theorists have suggested that horizontal transmission would be favored in a rapidly changing environment (Cavalli-Sforza et al. 1982).

One growing debate in the field of social learning is whether teaching occurs in small-scale societies, including among foragers. The human propensity for language, overimitation, and prosociality are all necessary for effective teaching, which some believe to be a uniquely human adaptation, essential to the evolution of cumulative culture (Dean et al. 2012; Gergely and Csibra 2006; Kline 2015; Tomasello et al. 1993; but see Caro and Hauser 1992 for examples of teaching in nonhuman animals). And yet, not all agree that teaching occurs across human cultures. Sociocultural anthropologist Lancy (2010: n. 1) defines teaching as “the active and systematic intervention of a teacher whose goal is to change the behaviour of a learner.” This definition closely resembles classroom teaching in a Western setting, and Lancy ultimately concludes that this kind of teaching does not exist in small-scale societies. Using this definition, MacDonald’s (2007) review of foragers learning to hunt also argued that teaching rarely occurs. And yet Kline (2015) demonstrates that teaching has been variously defined depending on the research field in question. A more functional definition derived from ethological studies defines teaching as the process an individual uses to modify their behavior for the benefit of facilitating another’s learning (Kline 2015). Therefore, importantly, teaching comes at a cost to the teacher (Caro and Hauser 1992). Under this definition, behaviors such as chore assignment, commands, and positive and negative feedback would be considered teaching, whereas under Lancy’s definition they would not. Indeed, using this more functional definition, various authors, exploring small-scale agricultural and foraging societies, have found evidence for teaching (e.g., Boyette and Hewlett 2017; Hewlett and Roulette 2016; Hewlett et al. 2011; Kline et al. 2013).

After considering the research on learning presented above, the present paper systematically compares previous findings on how children learn subsistence skills in forager societies. Since foragers are culturally distinct from other small-scale societies (Hewlett et al. 2000) and since our evolutionary history has largely been a foraging one (Marlowe 2005), focusing on foragers can provide us with unique insights into the contributions of learning on the evolution of modern human life history. Furthermore, of those studies focused on the association between the human life history strategy and learning in foragers, few have employed a cross-cultural approach, which allows us to draw broader trends from the literature. For example, Bliege Bird and Bird (2002), studying Meriam foraging, found that children made optimal foraging decisions based on their size, and thus size and not learning could explain their differing foraging returns. On the other hand, Walker et al. (2002) found that it takes Ache men more than 35 years to become proficient hunters, despite the fact that peak strength and size is reached in their twenties. Is methodology, environment, or culture the cause of these differences? Without a cross-cultural, comparative approach, it is difficult to say. Furthermore, hunting is not the only skill that is complex: toolmaking, for example, can also take a lifetime to master (e.g., Jordan 2014). And yet, no studies consider these skills through a life history framework. Thus, a broader approach to studying skill acquisition in general, as opposed to particular skills, is warranted. The present study aims to address both of these gaps by comparing cross-cultural data and studying skill acquisition as part of life history.

## Methods

Meta-ethnographies are primarily used to synthesize qualitative data for medical research, but they have important applications across various fields (Britten et al. 2002; Campbell et al. 2003; MacEachen et al. 2006). As with a systematic literature review, meta-ethnographies allow researchers to extract common themes and findings from studies from a variety of fields. However, unlike a systematic literature review, a meta-ethnography allows for the inclusion of both qualitative and quantitative studies so that our results may encompass a broader, more interdisciplinary range of publications.

### Search Strategy

The electronic databases used for this search included PsycInfo, JStor, Springer, Wiley, and ScienceDirect. We identified books and book chapters using the above search engines as well as Google Books and the Cambridge University library search system, which has referenced every book published in the UK. We found unpublished theses and dissertations using ProQuest. Our search terms paired the words “forager” OR “hunter-gatherer” with “child” and with “learn” OR “transmission” OR “socialization” OR “skill acquisition.”

In an effort to identify and include older anthropological publications on learning, we also surveyed the electronic Human Relations Area Files (eHRAF) World Cultures (ehrafworldcultures.yale.edu) online as of January 2016. We limited our search to those societies HRAF staff codes as hunter-gatherers and “primarily hunter-gatherers.” Then we searched for ethnographic passages coded by eHRAF staff as “socialization” (OCM code 860), “infancy” and “childhood” (OCM code 850), “learning behavior” (modification of behavior; OCM code 153), and “learning process” (ethnopsychology; OCM code 828) from the *Outline of Cultural Materials* (Murdock et al. 2008). eHRAF provided us with a list of papers that mentioned learning. We investigated each to determine whether they contained significant emphasis on hunter-gatherer learning in childhood.

We designed the final steps of our search in hopes of finding studies that we may otherwise have missed. First, we searched the references of relevant articles and book chapters. Second, we searched the references of qualitative literature reviews on learning in hunter-gatherer children. Third, we searched the publication lists of first authors of relevant publications. Fourth, we contacted the first authors of relevant publications. We provided them with our publication list, to ensure that we were not missing key texts, doctoral dissertations, or unpublished manuscripts. We also contacted all authors who contributed to the *Cambridge Encyclopedia of Hunters and Gatherers* (Lee and Daly 1999) for any published or unpublished manuscripts on learning in their study communities. Finally, we sorted the studies into two overall groups: studies on learning social skills and gendered behaviors (Lew-Levy et al. 2017) and studies on learning subsistence skills. This paper focuses on the latter topic.

### Eligibility Criteria and Study Selection

We included studies based on three criteria. First, that the societies in question were hunter-gatherers. Second, that the studies had primarily focused on learning. Third, that the studies considered the learning of children.

Academic definitions of hunter-gatherers have varied broadly over the years, with some focusing on a social definition of small-scale, egalitarian societies; others on a pure economy of foraging; and others still on the importance of mobility. For each of the various definitions of hunter-gatherers, the diversity of foragers across the world means there is always a group that will not fit (Kelly 1995). We chose to focus on socially defined small-scale, relatively egalitarian and traditionally foraging societies. There are no foragers today who do not accept economic input from domesticated plants and animals. Thus, we included groups such as the Penan, who sometimes participate in rice swidden agriculture, and the Aka, who trade with farming neighbours. Because of our focus on a social definition of small-scale foragers, we excluded some groups who, economically, are purely foragers, but whose societies are highly stratified. For example, we excluded North America’s Pacific Northwestern Kwakiutl, Nootka, and Makah, who subsisted entirely on wild foods, including plentiful salmon runs, but also held slaves. In considering the inclusion of studies on native North Americans and Australians more broadly, we exercised our judgment. Many of these cultures are, of course, foundationally foraging ones, though they have been forcibly removed from that lifeway. For this reason we included studies of Indigenous socialization or mid-century ethnographies of Native Americans that discuss children’s learning. We would like to note here that we included studies of foragers who attended school, as long as these studies focused on children’s foraging activities. The reasons for this are twofold: first, not all studies noted the degree to which children were formally educated. Second, two studies included in this review noted that years spent in school did not significantly influence children’s foraging performance (Blurton Jones and Marlowe 2002; Kawabe 1983). However, we specifically excluded any studies about learning in school. And, where we feel schooling might have influenced the results of a study, we explicitly address this topic (e.g., Nielsen and Tomaselli 2010; Nielsen et al. 2014a, 2016). Finally, many foraging groups are not represented in this study, and that may well be because there are no relevant studies about that group, not because we do not consider them to be foragers.

In this review, we only included studies that focused specifically on learning subsistence skills, or the processes associated with subsistence skills. We did not include studies that mentioned learning but did not specifically explore this topic. However, in older publications retrieved from eHRAF, mostly early- to mid-twentieth-century ethnographies, sections entitled “childhood” sometimes include detailed descriptions of socialization practices. These were included. We included studies that the authors in question define as focused on childhood. We also expected those studies we selected for inclusion to have at least some original data. These include studies that use various qualitative ethnographic methods (interviews, participant observation, etc.), experimental designs, quantitative behavioral observations, and quantitative interview techniques. We excluded studies that rely entirely on secondary data we could access elsewhere. However, we used the references from these studies to find their primary sources wherever possible. Finally, we excluded conference proceedings, as well as publications in a language other than English.

### Data Extraction and Synthesis

Data collection took place between January and March 2016. We extracted three types of data for each study included in this survey. (1) Descriptive data: the hunter-gatherer group(s) surveyed, the age group(s) surveyed, and the year in which the paper was published. (2) Methodological data: the objective of the study and the study design (interview, participant observation, behavioral observation, etc.). (3) The findings of the study in relation to the three questions of interest: How do hunter-gatherer children learn subsistence skills? How long does it take to learn these various skills? From whom do children learn these skills? As in all meta-ethnographies, in order to synthesize our findings, we organized the results according to themes that appear common in the literature.

## Results

### Descriptive Statistics

**Fig. 1:**
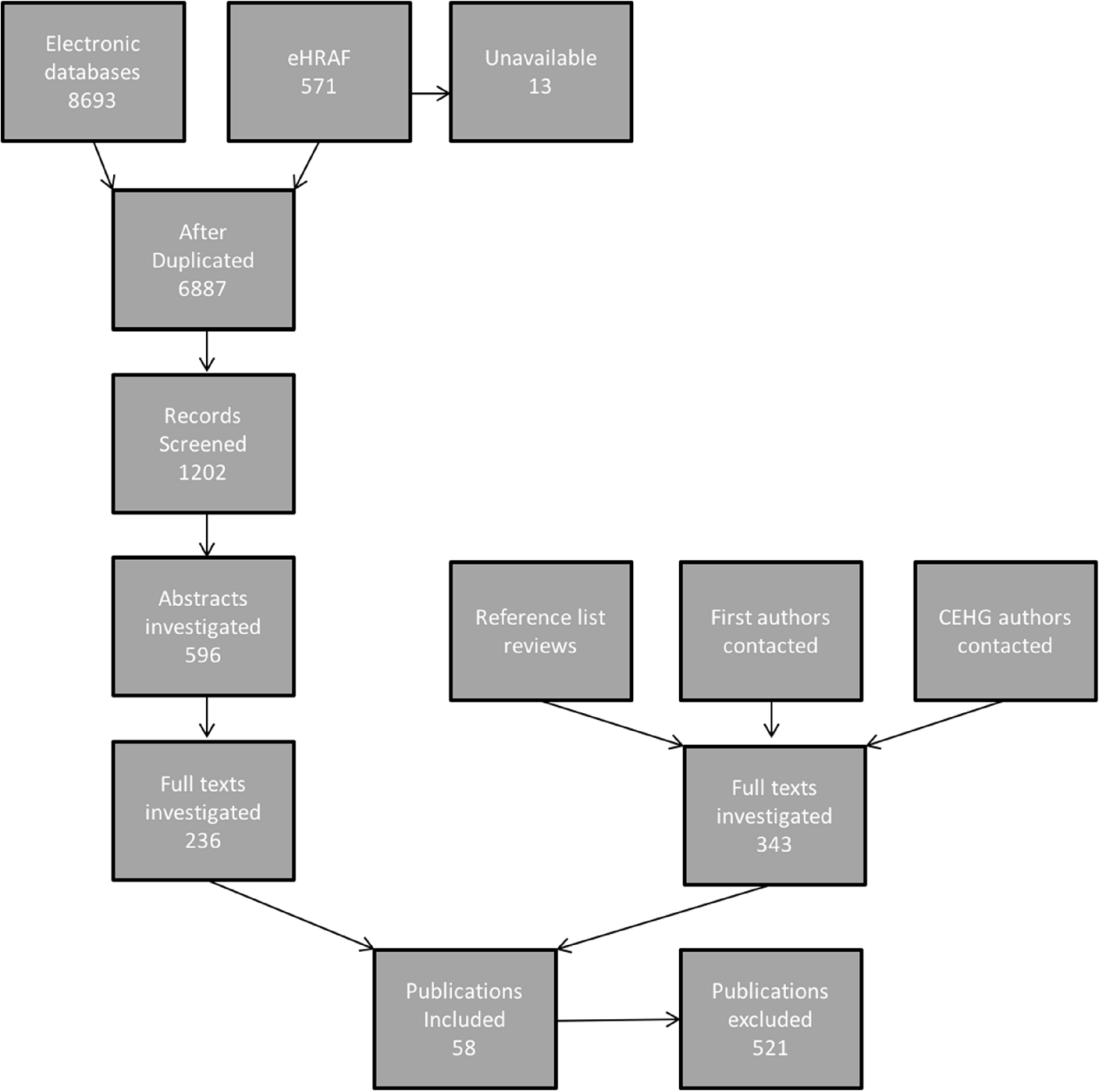
Flow chart of the publication retrieval procedure

Our initial keyword search, after eliminating duplicates, yielded 1202 potential studies (Fig. 1). We discarded 966 of these after screening the titles, publication type, and abstracts, and we selected 236 studies for full-text reading. From those studies meeting our criteria, we searched their references for relevant publications and contacted 60 first authors (we could not locate 4 email addresses), half of whom responded. We also contacted 37 contributors from the *Cambridge Encyclopedia of Hunters and Gatherers* (we could not locate 14 email addresses), of whom 9 responded. We also examined the references from six relevant reviews (Bugarin 2008; Eickelkamp 2010; Herzog 1974; Hewlett 2014; Keith 2008; MacDonald 2007). This yielded another 340 publications for full-text reading. The 58 publications that provided information addressing our three questions on learning subsistence skills were included in the present study.

**Fig. 2:**
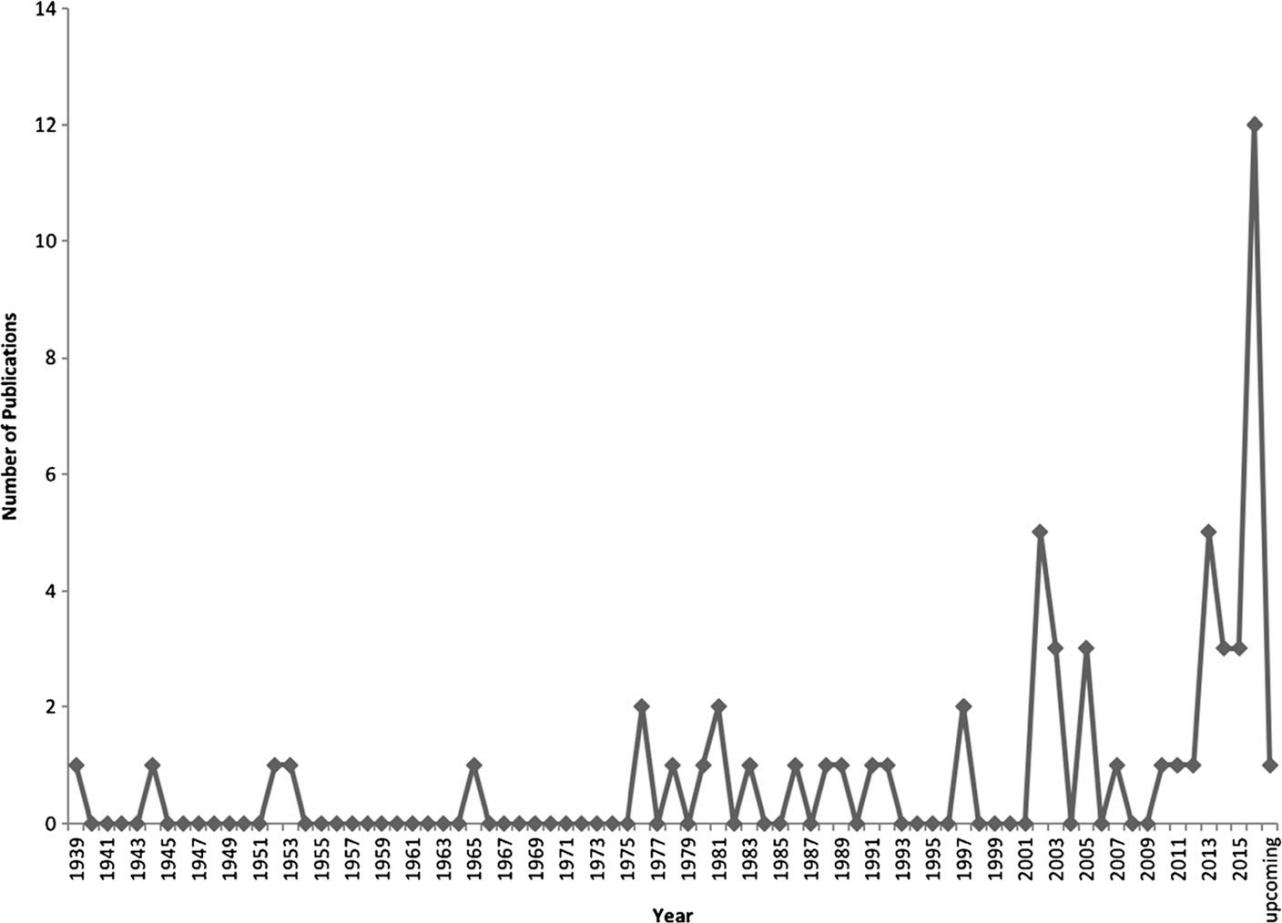
Number of publications per year, based on publications included in the present review

Of the 58 publications that we included, 7 (12%) use experimental data to answer their questions, 5 (9%) use narrative accounts of learning, 30 (51%) use quantitative data, and 33 (57%) use qualitative data.^1^ The earliest publication in our list is from 1939, with the great majority (39 vs. 19) being produced after the year 2000 and particularly in the past 5 years (2010–2015; 26 papers) (Fig. 2). Our list includes studies on 34 different cultures—plus two general studies of Australian Aboriginals—from five continents (Table 1).

**Table 1:** Contributing authors and number of studies included in the review by culture and continent

Country	Culture (*n* of studies)	First Author
Africa		
Botswana/South Africa	San (9)	Draper, Imamura, Shostak, Nielsen
Cameroon	Baka (3)	Gallois, Sonoda
CAR	Aka (11)	Neuwelt-Trunzer, B. S. Hewlett, Boyette, B. L. Hewlett, Berl, Fouts
CAR	Bofi (1)	Fouts
DRC	Efe (1)	Morelli
Ethiopia	Chabu (2)	B. L. Hewlett, Dira
Madagascar	Mikea (1)	Tucker
Republic of Congo	Mbendjele (1)	Lewis
Tanzania	Hadza (2)	Blurton Jones, Crittenden
Asia		
India	Jenu Keruba (1)	Demps
India	Nayaka (1)	Naveh
India	Ongee (1)	Pandya
Malaysia	Batek (1)	Lye
Malaysia/Borneo	Penan Benalui (2)	Puri
Sibera	Khanty (1)	Jordan
Siberia	Yukaghir (1)	Willerslev
Australia and Oceania		
Australia	Indigenous (not specified) (2)	Nielsen
Australia	Kaytetye (1)	Thompson
Australia	Mardudjara (1)	Tonkinson
Australia	Martu (1)	Bird
Australia	Meriam (2)	Bird, Bliege Bird
Australia	Pitjantjatjara (1)	Ilyatjari
Australia	Yolngu (1)	Harris
Papua New Guinea	Gidra (3)	Kawabe, Ohtsuka, Nishiaki
North America		
Canada	Chippewayan (1)	Vanstone
Canada	Cree (1)	Ohmagari
Canada	Montagnais (1)	Burgesse
Canada	Dene (1)	Gardner
USA	Comanche (1)	Wallace
USA.	Gros Ventre (1)	Flannery
USA.	Sioux (1)	Erikson
South America		
Paraguay	Ache (1)	Walker
Peru	Matsigenka (1)	Johnson

Two studies (one by Nielsen and one by Fouts) discussed more than one culture and are counted twice in this table. Nielsen included both the San and Aboriginal Australians. Fouts included both Aka and Bofi foragers

Our team identified five themes to organize our results: learning methods, learning to harvest and to hunt and trap small game, learning to hunt big game, learning to make material culture, and the impact of strength and skill on the age of skill acquisition. 18 studies (31%) explicitly focus on three learning methods: teaching (11 studies), overimitation (4 studies), and innovation (3 studies). 37 studies (64%) focus on children learning to gather and to hunt and trap small game. The authors argued that same-sex vertical transmission (8 studies), observation (15 studies), play (15 studies), and participation (20 studies) are especially common ways to learn these skills, and thus we outline each of these separately. 10 studies (17%) discuss learning to hunt big game. 11 studies (19%) focus on how children learned to make material culture. Finally, 5 studies (9%) focus on determining whether strength, skill, and experience are factors in the age of skill acquisition.

### Learning Processes

Though the process of learning is widely discussed in the publications, some works are more specifically focused on learning processes such as teaching, imitation, and innovation. We address those specialized papers here.

#### Teaching

When teaching is not limited to Western-style direct instruction but is defined to include demonstration, commands, and positive and negative feedback, many authors have found that teaching does play a role in forager children’s learning. In fact, a series of four studies based on systematic behavioral observations of the Aka found, unsurprisingly, that the Aka style of teaching is qualitatively different from teaching in WEIRD societies (Boyette 2013; Boyette and Hewlett 2017; Hewlett and Roulette 2016; B. S. Hewlett et al. 2011). In addition, these authors found that, among the Aka, teachers are more likely to be biologically related to the learner in question, and that mothers are the most significant contributors to teaching. Specifically, Boyette’s (2013; Boyette and Hewlett 2017) cross-cultural study of teaching among Aka foragers and Ngandu farmers found that direct instruction does occur among both groups, but it is significantly more common among the Ngandu. Boyette also found that commands are the most frequent form of teaching for both groups, though the Ngandu tend to be commanded specifically to perform work tasks, whereas Aka children are commanded to perform behaviors across various skill domains. Negative feedback is the next most common form of teaching for the Aka and is usually in response to breaches of social norms, especially sharing (Boyette 2013). Finally, in the case of children teaching other children, children are most likely to be taught by those older than them, and by same-sexed children (Boyette and Hewlett 2017).

Another broad theme across many ethnographic studies is the importance placed on children’s autonomy in their own learning process, meaning that adults prefer to allow children to observe and experiment with minimal interference. Among people as broadly ranging as the San (Draper and Cashdan 1988), Nayaka (Naveh 2014), Batek (Lye 1997), Matsigenka (Johnson 2003), and Yukaghir (Willerslev 2007), adults actively refrain from instructing, directing, explaining, or correcting, valuing firsthand knowledge gained by the child through personal experience over any kind of secondhand knowledge. Learning is therefore characterized by processes of trial and error and is embedded in the context of living in close quarters, and having the opportunity to observe others through everyday tasks and conduct (Naveh 2014). Among the Dene, individual autonomy and learning in childhood are not competing aims, with children actively provided with opportunities to watch an especially careful but silent version of a task, rather than explicit instruction (Christian and Gardner 1977). And although the Dene consider paying attention critical to learning, at no point do they insist that the learner pay attention. Similarly, Draper (1976) described a scene in which an adult was stretching a hide. Next to him, a child watched his actions intently, but the man did not change his behavior to accommodate the child. Children in these contexts initiate their own learning; experiment with objects, bodies, and feelings; and adjust their behavior according to the results of their actions. Christian and Gardner (1977) and Naveh (2014) both argued that such learning leads to diverse understandings, with no attempt to form a systematic and unified standard form of either social or practical knowledge. Similarly, Lye (1997) highlighted that among the Batek, though instruction does occur, personal experience of moving in the forest, monitoring one’s own skills, and training one’s body is considered the best way of acquiring knowledge.

#### Overimitation

Though imitation is a common form of children’s learning across the world, researchers have recently become interested in how culture influences the frequency of overmitation as a way of pinpointing basic differences in how children learn. Using experimental designs, many studies have found overimitation to be common in WEIRD children, but among hunter-gatherer children the results are more mixed. Four studies exist that are specifically on foragers, and all use puzzle boxes in their experimental design (Nielsen et al. 2016; Nielsen and Tomaselli 2010; Nielsen et al. 2014a). Nielsen and colleagues have conducted four studies on overimitation comparing Brisbane preschoolers, San hunter-gatherers from Botswana and South Africa, and/or Australian Aboriginal children, all ranging in age from two to six. They found that, across the board, hunter-gatherer children overimitated at the same frequency as Brisbane children. In contrast with these findings, Berl and Hewlett (2015) found that Aka children ranging in age from four to seven engaged in overimitation far less than Ngandu farmer children of the same age, and less than Aka adults, though all participants were more likely to perform the irrelevant actions than not.

#### Innovation

Three studies specifically on forager children’s ability to innovate suggest that innovative behaviors do not fully emerge until adulthood, but that these innovations are then transmitted primarily to adolescents. Nielsen et al. (2014b) used an experimental design to determine whether South African San and Brisbane children between the ages of three and five could innovate new tools to fetch a toy from a bucket. The children had access to a multitude of tools, including a pipe cleaner that could be bent to retrieve the toy. The results indicate that few children chose the pipe cleaner as their first tool. Half of the children were unable to innovate a tool to retrieve the toy. However, once these children were shown how to produce the tool—for example, shape the pipe cleaner into a hook—nearly all were capable of producing and using them. Thus, Nielsen argued that innovative behaviors are not yet fully developed in early childhood, irrespective of culture. Hewlett has also conducted a study of innovation among Chabu (Hewlett 2016) and Aka (Hewlett 2013) adolescents and found that, in both groups, adults were the key innovators. Adolescents sought prestigious innovators who could teach well, irrespective of how far away they lived. Furthermore, adolescents affirmed that they utilized innovations to find a mate, and also to provide for their families. According to Hewlett, adolescents are more likely to seek out innovative teachers than children or other adults, and these teachers are usually not their parents.

### Learning to Forage and to Hunt and Trap Small Game

For hunter-gatherers, learning subsistence skills begins early in life. In infancy, children accompany parents, especially mothers, on foraging expeditions, where they have ample opportunity to watch subsistence activities (Hewlett et al. 2011; Lye 1997). Children in infancy and early childhood also play with their parents’ tools, including potentially dangerous ones such as machetes (Hewlett et al. 2011; Lewis 2002; Lye 1997). Authors described parents making toy versions of fishing lines, baskets, digging sticks, spears, and bows and arrows for children across cultures, including the Gidra (Nishiaki 2013), Batek (Lye 1997), Kaytetye (Thompson 2003), Chabu (Dira and Hewlett 2016), Aka (Hewlett et al. 2011; Neuwelt-Truntzer 1981), Comanche (Wallace and Hoebel 1952), Hadza (Crittenden 2016), and the San (Imamura 2016). Among the Batek, by two years of age children are already learning ecological taxonomies (Lye 1997). By the age of six, Meriam, San, Batek, Chabu, and Pitjantjatjara children have an understanding of environmental hazards (Bliege Bird and Bird 2002; Dira and Hewlett 2016; Ilyatjari 1991; Imamura and Akiyama 2016; Lye 1997). These are learned from parents (Bird and Bliege Bird 2002) and through stories (Dira and Hewlett 2016). By adolescence, at the latest, various authors note that children are already competent food collectors, though they may refine more complex skills, such as hunting, throughout their adult life (Crittenden 2016; Dira and Hewlett 2016; Gallois et al. 2015; Hewlett and Cavalli-Sforza 1986; Lye 1997). The major ways that children learn varying foraging skills include same-sex vertical transmission, observation, play, and participation. We address the results for each of these learning mechanisms in turn.

#### Same-sex Vertical Transmission

Hewlett and Cavalli-Sforza (1986), Thompson (2003), Ilyatjari (1991), Flannery (1953), and Burgesse (1944), among others, have argued that children learn many foraging skills through vertical transmission from same-sex parents. For example, among the Gros Ventre, formal training for skills necessary to women’s work, such as collecting berries and digging roots, comes from female relatives (Erikson 1939). Among the Sioux, mothers are the primary transmitters of food preparation knowledge, shelter building, and hide work to their daughters (Flannery 1953). For the Aka, parents are the primary transmitters of food acquisition skills, with fathers generally transmitting skills to their sons, and mothers, to their daughters (Hewlett 2012; Hewlett and Cavalli-Sforza 1986). For example, Aka men know more than women about hunting, and therefore fathers contribute more to the acquisition of those skills. Demps et al. (2012) argued that Jenu Keruba fathers are also particularly important in transmitting knowledge about honey collecting—an activity typically performed by men—to sons between the ages of six and nine.

#### Observation

Observation appears to be central to how forager children establish competency in many foraging tasks while still very young (Boyette 2013; Burgesse 1944; Draper 1976; Flannery 1953; Harris 1980; Imamura and Akiyama 2016; Ohmagari and Berkes 1997; Tonkinson 1978; Vanstone 1965). For example, Morelli et al. (2003) noted that Efe children between the ages of two and three spent a quarter of the authors’ scan observations observing work. Indeed, Neuwelt-Truntzer (1981) argued more generally that Aka children spend much of their time simply watching all adult activities. Children are, after all, almost constantly in view of adults, particularly when they are very young (Draper 1976; Hewlett et al. 2011; Lye 1997). Naveh (2014) noted that, among the Nayaka, children watch adults set traps and then simply practice trap setting themselves. Jenu Kuruba adolescent boys learn to make smoky torches and cut honeycombs by observing older kin as they collect honey from locations too dangerous or difficult for the children to actually participate in the process (Demps et al. 2012).

#### Play

The authors we include emphasize play as a crucial method for children to learn foraging skills. According to Morelli et al. (2003), Efe children spend significantly more time emulating adult activities in play than American children. Boyette (2013, 2016) and Gallois et al. (2015) found that, as children grow older, they play less and work more, suggesting that play helps them learn subsistence behaviors. In comparing Aka and Bofi foragers with Bofi farmers, Fouts et al. (2016) found that, though Bofi farmer children between the ages of one and four participated in more work-themed play than their foraging counterparts, forager children were in closer proximity to adults and were more likely to use objects when performing work-themed play. Parental beliefs may contribute to cultural beliefs with regard to play; Neuwelt-Truntzer (1981) argued that Aka parents believe that if children do not play, they will fail to learn. Among a vast cross-cultural sample including the Mbendjele, Hadza, San, Katetye, Aka, Mardudjara, Pitjantjatjara, Chippewayans, and Gros Ventre, children build small huts and hearths (Crittenden 2016; Flannery 1953; Ilyatjari 1991; Lewis 2002; Neuwelt-Truntzer 1981; Shostak 1976; Thompson 2003; Tonkinson 1978; Vanstone 1965). Near these huts, children pretend to dig yams, to hunt, and pretend to be animals. Through these kinds of games, children also learn human-animal relationships. Naveh (2014) suggested that children who play hunted animals in such games vocalize the animal’s feelings, fears, and emotions. Through this activity, children learn to sympathize with animals and to see animals as sentient persons sharing the forest world with them.

#### Participation

Children do not just observe their parents’ subsistence activities; they also learn through participation. In fact, among the Aka, Neuwelt-Truntzer (1981) noted that children may be included in any adult activity. Hewlett (2014) also noted that Aka girls learned to forage by walking in the forest with their parents. Sonoda (2016a, 2016b) described adults acknowledging Baka children when they enter situations where hunting and gathering is taking place, and giving the children access to resources. Both adolescents and adults help children learn through participation by providing them with verbal instruction and other subtle forms of teaching. According to Dira and Hewlett (2016), Chabu adults allow children and adolescents to participate in the killing of animals. Vanstone (1965) mentioned that Chippewayan children learn adult skills and attitudes by participating directly in the household economy. From early childhood onward, Baka children are also expected to participate in household chores, such as fetching water and firewood (Gallois et al. 2015). Among the Cree, women report hands-on experience as the primary way they learn a variety of skills as children and adolescents, including fur preparation, food preparation, camp-related skills, hunting, fishing, and trapping (Ohmagari and Berkes 1997). That being said, Draper and Cashdan (1988) found that the work of San parents, such as nut cracking, is more efficiently done by adults, and the nature of this work can make it difficult for children to participate.

At times, however, children participate in adult activities without adults being present, shifting the locus of learning to child-to-child knowledge transmission. Neuwelt-Truntzer (1981) noted that in middle childhood, children participate in work groups in which they display self-reliant behaviors such as food harvesting. Indeed, Crittenden (2016) highlighted the importance of “learning by doing” that occurs within children’s playgroups. Crittenden (2016), Lewis (2002), and Gallois et al. (2015) described children collecting wild foods and roasting them on their own hearths. In fact, Crittenden (2016) argued that children are the only Hadza who harvest weaverbirds, a skill primarily transmitted within the playgroup. Among the Meriam and Martu, details and strategies for foraging are learned through other children, and children make decisions to optimize their foraging returns based on their size and strength (Bird and Bliege Bird 2002, 2005; Bliege Bird and Bird 2002). Similarly, Tucker and Young (2005) noted that Mikea children allocate as much time to foraging as do adolescents. Thus foraging emerges as an extension of play. For example, they described children harvesting tubers (work), and then having a food fight (play) with those same tubers. Gallois et al. (2015) also highlighted that though children are not expected to participate in economic activities, they do so out of enjoyment. Jenu Kuruba children learn to climb trees to collect honey through games played with their peer groups (Demps et al. 2012). Through these playgroups, older children also transmit early hunting skills (Crittenden 2016; Hewlett et al. 2011; Imamura 2016; Imamura and Akiyama 2016; Thompson 2003). It is through older children that San children learn how to bait traps, for example (Imamura 2016; Imamura and Akiyama 2016). Through peer group participation, Baka children learn to identify edible wild plants, navigate the landscape, and use increasingly complex tools (Gallois et al. 2015).

### Learning Big-Game Hunting

Hunting is one of the most difficult skills that children, primarily boys, learn. Though children seem to become proficient at small-game hunting relatively early in life, big-game hunting may require a lifetime to master. At first, much of this learning process takes the form of translating observed adult activities into organized games played with peer groups. A hide-and-seek game played by the Ongee, for example, helps children develop the skillset to find animals hiding in the bush (Pandya 1992). Among the Chabu, children play collaborative role-playing games of hunter and hunted (Dira and Hewlett 2016). Similarly, Nisa, a San woman, described playing at hunting during her childhood (Shostak 1976, 1981). Nisa and her friends followed tracks, and when they spotted prey, they shot make-believe arrows at them. Then, they took leaves and put them on a stick, pretended it was meat, and carried it back to the village. Among the Mbendjele, Pitjantjatjara, and Kaytetye, spear-throwing games and other target practice, such as boomerang competitions, are important for developing accuracy (Ilyatjari 1991; Lewis 2002; Thompson 2003). Similarly, according to Wallace and Hoebel (1952), peer-group learning is central to Comanche children’s development of shooting accuracy.

Yet hunting seems also to be one of the most prominent exceptions to the general lack of direct instruction among hunter-gatherers, likely because of the complexity of hunting. And, in several cases, direct instruction in hunting-related skills begins in early childhood. Around the ages of six or seven, Chabu children listen to hunting stories by their fathers (Dira and Hewlett 2016). These stories transmit important information regarding animal sign and behavior, as well as dangers associated with hunting. Batek children learn to imitate animal sounds by age six, and they regularly practice dart hunting by age nine (Lye 1997). Before adolescence, Batek children are already proficient at hunting birds and squirrels.

During adolescence, children in many cultures receive prominent direct instruction in hunting skills. Among the Chabu, Dira and Hewlett (2016) recorded observation, demonstration, verbal instruction, pointing, and teasing as important teaching processes when adolescents are learning to hunt from their mentors. For the Penan, for whom extensive speaking in the forest is taboo, teachers help children learn to hunt by pointing and describing actions, by providing children with opportunities to watch hunting, and by imitating bird and animal calls (Puri 2005). Among both the Chabu and the Batek, boys choose their hunting teachers (Dira and Hewlett 2016; Lye 1997). They trail these hunters and are tutored by them. Chabu adolescents choose teachers based on their hunting ability, skill as teachers, or knowledge of ecology. Chabu adolescents primarily learn to spear hunt from other adults as well as peers, beginning between the ages of nine and twelve. For Penan boys (and sometimes girls), fathers, uncles, and other elders are the primary teachers of hunting skills between the ages of four and fourteen; older boys between ages 14 and 20 learn hunting with peers (Puri 2005). Wallace and Hoebel (1952) argued that Comanche grandfathers specifically are heavily involved in teaching their grandsons to ride horses, shoot, and hunt.

### Learning to Make Material Culture

Studies of how children learn to produce material culture seem to demonstrate that such skills are transmitted mostly vertically, from parents to offspring, and also commonly from older children. As one might expect, in many cases children begin to learn craft skills by making small-scaled versions of items such as bows, arrows, and sledges. In a study of how Baka children spend the majority of their time, Gallois et al. (2015) determined that they participate in subsistence and leisure activities more frequently than in handicrafts. This trend generally holds true among the publications included here; that is to say, hunter-gatherers do not seem to emphasize structured instruction on creating material culture, especially among their younger children.

During early and middle childhood, children continue to learn from models, and the role of other children in their learning process becomes more prominent. Between the ages of four and five, Batek (Lye 1997), San (Imamura 2016; Imamura and Akiyama 2016), and Kaytetye (Thompson 2003) children begin making their own tools. In these cases, parents gift children with bows and arrows while they are still too young to use the tools, let alone to produce them. Among the Batek (Lye 1997), parents correct children’s mistakes on tool construction; among the San (Imamura 2016; Imamura and Akiyama 2016) and Kaytetye (Thompson 2003), younger children imitate older children to learn how to construct these tools, and they are also corrected by other children. By ages four and five, San and Batek children have constructed the bows and arrows they will use to hunt birds and lizards until adolescence (Imamura 2016; Lye 1997). Nishiaki (2013) argued that Gidra parents intend their gifts to be a form of education. Rather than directly teaching children how to produce bows and arrows, parents gift them with well-made scale models from which they are expected to reverse-engineer their own tools. This may also be true among the Aka, who made fragments of nets available to children so they can examine them (Neuwelt-Truntzer 1981). Gidra children do not skillfully produce bows and arrows until approximately 14 years old. On the other hand, Imamura and Akiyama (2016) argued that, after mothers first gift their two- to three-year-old sons with bows and arrows, the boys then refine their skills in bowmaking and in the hunting of small game largely with the help of older boys. Imamura (2016) emphasized the role of older San boys as well, stating that older children will take over and complete toys for younger children when they are struggling with the skill.

Direct instruction from adult to child in the production of material culture seems to be clustered later in childhood and in early adolescence, when children begin producing more complex material culture. Other handicraft skills, including basketry (Puri 2013), hideworking (Erikson 1939; Ohmagari and Berkes 1997), and the production of skis, sledges, and canoes (Jordan 2014), seem to be taught using vertical and oblique transmission in late childhood to early adolescence. Jordan (2014) argued the Khanty transmit skills such as ski, sledge, and canoe production vertically, and that children learn from observation, imitation, and direct instruction. When learning to construct sledges, children in late childhood create exact models of adult sledges. Cree women report learning hideworking skills between the ages of 11 and 16, mostly from hands-on experience and family instruction (Ohmagari and Berkes 1997). Sioux hideworking is learned early on, primarily from mothers (Erikson 1939). Among the Penan, Puri (2013) found that women report beginning to learn basketmaking at age 14, on average, whereas males begin somewhat later, at 17. However, he acknowledged that among some families, for whom basket making is especially important, children begin to learn as early as age eight. Because men and women make and use different baskets, boys tend to learn from men and girls from women, usually family members but not necessarily parents.

### Strength, Size, Skill and Experience in Foraging Proficiency

Though this review is primarily concerned with learning in childhood, we include studies concerning how body size and strength as opposed to skill and experience can impact foraging proficiency. Children’s learning processes are, after all, framed by their size and their relative lack of experience. Overall, these studies find that the more complex the activity, such as hunting in particular, the more important experience may be. Walker et al. (2002), working with the Ache, conducted an experimental and quantitative observational study on individuals ranging from 12 to 40+ years of age. The authors found that prey finding rates peak in the late thirties, as do hunting abilities. However, ability to hunt monkeys, one of the most difficult prey in the Ache ecosystem, peaks in the forties. Walker et al. (2002) also found that previous lack of experience adversely affects hunting ability. Similarly, Ohtsuka (1989), working with the Gidra, found that, independent of strength and size, individuals between the ages of 35 and 45 have four times the hunting success of teenagers and young adults. These two studies suggest that strength is less important than skill in hunting proficiency. Kawabe (1983) found that Gidra boys hunt a larger variety of animals as they grow older. These expand from small animals, which are easy to hunt, to larger animals, which can be hunted with developed skills. Though older boys vary in success rates, Kawabe suggested that this is related to differences in environmental knowledge, not arrow shooting proficiency. Finally, Hewlett and Cavalli-Sforza (1986) found that though Aka girls and boys between the ages of seven and twelve have developed a majority of their foraging skills, only the boys will continue to increase their skills in net hunting and other hunting techniques through adolescence and adulthood.

Other individual components of hunting activity, such as shooting accuracy, seem to require less experience to achieve proficiency. And some simpler foraging activities, such as tuber digging or tree climbing, require a baseline of strength, after which increased experience does not significantly improve returns. In an experimental study with the Hadza, Blurton Jones and Marlowe (2002) considered the importance of practice in proficiency at tree-climbing, target shooting with bows and arrows, and tuber digging through an “Olympics”-style competition, including children, adolescents, and adults of both sexes. The authors found that women and men were equally proficient at digging tubers, despite the fact that women had significantly more experience doing so. Similarly, the authors found that adolescents who attend boarding school were just as proficient at climbing trees and just as accurate in shooting as their unschooled peers, despite having practiced these skills less. Kawabe (1983) also found no remarkable difference between schooled and unschooled Gidra boys in some foraging tasks, possibly because schooled boys take advantage of hunting opportunities when they return to the village during long vacation.

## Discussion

These results indicate a meta-ethnographic approach has utility for answering the kind of broad ethnographic and evolutionary questions we have posed here; how do children learn subsistence skills, from whom do they learn them, and how long does it take to reach proficiency? In recent years, a growing number of researchers have been interested in these questions. However, this interest is unevenly distributed, with the San and the Aka receiving the most consistent attention on learning in childhood. This is likely due to the interests of researchers such as Patricia Draper and Barry and Bonnie Hewlett, who have contributed immensely to the field of learning in hunter-gatherer childhood. However, this represents an African bias in the literature. More studies are needed on learning in childhood among foragers on other continents.

Nonetheless, taken cumulatively, the studies demonstrate that social learning occurs before individual learning among hunter-gatherers, which aligns with what several authors have predicted to be the most adaptive progression of learning. Our results also emphasize the importance of observation, participation, and same-sex parental transmission in learning subsistence skills. In particular, the playgroup and playful learning allow forager children to take increasing responsibility for provisioning themselves (though they do not always do so) without considering subsistence activities to be a burden. Our results clearly show that teaching exists among hunter-gatherers in the form of feedback and demonstration. Direct instruction appears to be largely reserved for adolescents, and for complex skills such as hunting and multicomponent toolmaking. We have found that adolescents are not innovators, but they are the primary acquirers of innovative behaviors. And, finally, our results suggest that while innovation may not explain our extended childhoods, children do spend their entire childhoods learning the complexities of hunting in particular. They do not, however, require an entire extended human adolescence to become proficient foragers of many plants and small game. In order to unpack our results more fully, we address the following points in our discussion: (1) Does teaching, overimitation, and innovation occur during hunter-gatherer childhood? (2) How and from whom do children learn? and (3) Does it take 20 years to learn to hunt and gather?

### Teaching, Overimitation, and Innovation

In the debate about teaching among hunter-gatherers, our results demonstrate a stark divide between ethnographic studies, which generally argue against the presence of teaching, and quantitative approaches, which find that it does occur. We would argue this debate is largely the result of a lack of consensus about the definition of teaching itself. We support Kline’s (2015) integrative definition of teaching, which includes the following behaviors: teaching by social tolerance, teaching by providing opportunities, teaching by stimulus or local enhancement, teaching by evaluative feedback, and direct, active teaching. Using this broad definition, we argue that each of these teaching styles exists to varying degrees in hunter-gatherer populations. For example, teaching through local enhancement occurs when children help butcher an animal (e.g., Dira and Hewlett 2016). Teaching through evaluative feedback occurs when parents correct children’s toolmaking (e.g., Jordan 2014). When children actively watch an adult tanning a hide, they are experiencing social tolerance (e.g., Draper 1976). Direct, active teaching also seems to occur, but is rare, and is most commonly used in adolescence to learn skills such as hunting and complex tool making (e.g., Dira and Hewlett 2016). However, even where direct teaching does occur among hunter-gatherers, it is qualitatively different than classroom teaching. It is specific to context—such as being out on a hunt—and depends on the child’s willing participation. Because the current teaching debate seems to hinge so heavily on semantics, we hope that researchers will adopt a more holistic definition like Kline’s, which would foster interdisciplinary conversation on the topic.

The varying results we report here on overimitation, with San and Aboriginal children found to overimitate much more prominently than Aka children, may be the result of compulsory Western schooling. The San and Aboriginal groups studied by Nielsen et al. (2014a) have access to classroom-based schools (Berl and Hewlett 2015). The Aka children studied did not. Children quickly learn to defer to teachers in a school setting and thus are more likely to imitate adults’ relevant and irrelevant actions. Indeed, some studies suggest that children generally are more likely to copy adults than they are to copy other children (Wood et al. 2012; Zmyj and Seehagen 2012). Among hunter-gatherers, though, autonomy and egalitarianism reduce the degree to which any individual defers to another based on age, gender, or status (Lewis 2014; Woodburn 1982). Since schooling often acts as a tool to incorporate marginal groups into the dominant culture, it seems likely that not only cultural values, but also learning processes, change (Berl and Hewlett 2015; Mesoudi et al. 2015). Further research into the presence of overimitation in foraging societies with differing access to schools could, therefore, provide important insight into how foraging children’s learning processes change. Furthermore, future research should also examine the degree to which social goals, such as group membership, and learning goals, such as proficiency at a given task, influence imitative behaviors (Over and Carpenter 2012, 2013).

Some have argued that the extension of childhood can be explained as an adaptation that provides children time to develop innovative behaviors (Bateson 2014; Carruthers 2002). Specifically, children’s play may be crucial to the development of the kind of human innovation that allowed anatomically modern humans to inhabit every ecosystem on the globe (Carruthers 2002). Yet, among modern hunter-gatherers our results do not support extended juvenility as time used for innovation. They do, however, potentially support the hypothesis that the skills learned in childhood create a foundation for future innovation during adulthood. Children cross-culturally do not appear to truly innovate, in the sense that they do not generally create new technologies or significantly different foraging methods for themselves (Hewlett 2013). Instead, our results suggest that forager children act as problem-solvers—using combinations of all of their knowledge in flexible iterations so they are prepared to truly innovate in adulthood (e.g., Naveh 2014). For example, Meriam children make their own decisions about resource choice, decisions that are couched in their background knowledge of the dangers and opportunities of the reef as a whole (Bliege Bird and Bird 2002). Indeed, Hewlett (2013) found that Aka adolescents seek out very skilled innovators to learn from, but they themselves do not innovate. Instead, children’s propensity for engaging in group social learning earlier in life and in innovation later on allows them to quickly gain a wide base of knowledge, which they can update with their own innovations as adults (Aoki et al. 2012).

### How and from Whom Do Children Learn?

Lehmann et al. (2013) argued that vertical transmission is most adaptive during infancy and early childhood, and that horizontal transmission and innovative, individual learning should occur throughout the rest of childhood. Our results support this pattern. In infancy we find that parents, not siblings, are primary caregivers (Draper and Cashdan 1988), and thus vertical transmission is common at this age. Many studies find same-sex vertical transmission to be especially important. Mothers teach their daughters gendered skills such as hide tanning, while fathers teach their sons to hunt. Parents have also been found to the primary transmitters of social skills, such as sharing (Boyette 2013). In early and middle childhood, both horizontal and oblique (child) transmission are important. Older children correct the tool manufacture of younger ones and show them how to bait traps (Imamura and Akiyama 2016). Play is also an important medium for horizontal transmission (Crittenden 2016). In adolescence, both oblique and vertical transmission are important for teaching and demonstrating more complex tasks, such as multicomponent tool manufacture and hunting (Dira and Hewlett 2016). Again, somewhat contrary to Lehmann et al.’s (2013) expectations, we do not find truly innovative behaviors emerging until after adolescence.

Specifically, our results emphasize the importance of children’s playgroups for learning subsistence skills, especially in middle childhood. Hunter-gatherer children are active learners who participate in learning by choice, and for whom learning is an ongoing, playful activity, not separated from the rest of life. Our results show again and again the prominence of what Gaskins and Paradise (2009) call “open attention,” a form of learning found in small-scale societies where children are in such constant contact with adults and older children as they work that learning occurs without the child or the “teacher” specifically intending it. In these contexts, learning may be an “incidental byproduct of social life” (Gaskins and Paradise 2009:85). This type of learning is exemplified by our findings that, cross-culturally, children continue to participate in foraging activities even when away from adults. This is markedly different from studies of small-scale farmers that emphasize a compulsory chore curriculum (Gaskins and Paradise 2009; Lancy 2012). It would seem that, through play, forager children can offset the cost of their burden of care, reducing the need for direct parental teaching.

This finding highlights Crittenden’s (2016) and Tucker and Young’s (2005) argument that play and work should not be distinguished since they are not distinguished by forager children themselves. Indeed, it would seem that, at least in the hunter-gatherer context, *both* play and work are a form of participation, and children transition seamlessly between the two. This finding supports arguments about the primacy of play in learning made by Bock and colleagues (Bock 2002, 2005; Bock and Johnson 2004), and the sociocultural approach to learning (John-Steiner and Mahn 1996; Lancy 2015). In his work with the Okavango Delta peoples, Bock (2005) found that children trade-off play with work, depending on the needs of the household and the complexity of the task at hand. For example, the playing at pounding grain allowed girls to practice this task without wasting grain, and boys’ participation in target games diminished as participation in actual hunting increased. These findings are supported by Boyette (2013, 2016) and Gallois et al. (2015), who also found that play and work traded off with age. Small-game hunting and trapping, which we found to be primarily learned in the playgroup, are excellent examples of these types of activities, wherein children can begin to assist in provisioning themselves while also learning important skills for later hunting of larger animals. Others, such as Göncü et al. (2006), suggest that play helps children situate themselves within a cultural world. Our findings that foraging children imitate the entire structure of adult subsistence activities through their play, such as building small huts and cooking their own foods on their own small hearths, supports Göncü’s hypothesis.

### Does It Take 20 Years to Learn to Hunt and Gather?

Yet another hypothetical driver for humans’ extended juvenility is our need for an extended period of learning (Kaplan and Robson 2002). So does it take 20 years for a modern hunter-gatherer child to learn to hunt and gather? Yes and no. In many ways, children are competent foragers by the end of late childhood, able to make simple tools, to gather plants and to hunt small animals, and even to make optimal foraging decisions about which resources they can most effectively exploit. However, the most complex skills of a hunter-gatherer’s life, such as big-game hunting, ecological knowledge, or the production of multicomponent tools, seem to be learned at the very latest stages of childhood and into adulthood. And these findings do not seem to be restricted to egalitarian foraging populations. Among the Tsimane, Bolivian forager-horticulturalists who fish and hunt extensively, a series of studies on hunting skills (Gurven et al. 2006; Schniter et al. 2015) argued that learning itself, not physical development or body size, seemed to determine hunting success. In fact, although Gurven et al. (2006) found that indirect encounters with game are most frequent in individuals’ mid-twenties, overall kill rates across multiple categories of game did not peak until age thirty-nine. Thus, the integration of all of a child’s individual skills into his or her maximum foraging potential may not occur until far past his or her transition into adulthood. This finding supports the theory that humans require a long juvenile period to learn to extract complex resources, though they do not need that long to learn all of their constituent foraging skills (Kaplan and Robson 2002).

## Conclusion

Through the years, more and more studies have focused on how foraging children learn subsistence skills. This meta-ethnography has allowed us to draw broad cross-cultural patterns from that positive trend in research. In infancy, children accompany parents on subsistence tasks and are given small versions of tools such as digging sticks and bows and arrows. During the transition from infancy to early childhood, when children join playgroups, they learn a majority of their subsistence skills, such as harvesting, trapping, small-game hunting, and some elements of honey harvesting, such as tree climbing. Children learn these skills through a variety of mechanisms, including participation in activities with adults and other children, through play, and via observation. It would seem that most children are proficient at these skills by the end of middle childhood. However, skills such as hunting, making complex tools, and learning innovative behaviors—skills that are more difficult and potentially more hazardous—continue to be learned into adolescence. These skills especially are learned obliquely, from expert adults, though parents seem to be prominent in teaching about material culture. The more complex the skill, the more common teaching seems to be. Finally, our results suggest that learning to hunt continues into adulthood.

A large-scale meta-ethnography will necessarily have limitations brought on by the sheer breadth of our sources, both in age and in methods. We did not include studies with general, passing mentions of learning, meaning that fragmentary observations in the literature are missing from this work. Many of the studies we did include only address positive observations without referencing the absence of specific behaviors, potentially introducing further bias. And, the relatively qualitative nature of our results and discussion means that we translated some quantitative results into qualitative findings, potentially misrepresenting their magnitude. Furthermore, many of the publications used different methods that are difficult to compare, especially as they were published over a 77-year timespan. However, because we are attempting to address extremely broad trends in hunter-gatherer behavior, it is our hope that these limitations are counteracted by the sheer quantity of data we include.

As we consider all of the papers included in this study, several general gaps in research become apparent. First, perhaps unsurprisingly, many papers focus on hunting activities; plant harvesting and other activities such as food preparation and childcare are given much less attention. In fact, we found no studies on how children learn to cook. Furthermore, studies on highly complex foraging activities such as medicinal plant use are lacking, and studies of the methods and timing of broad traditional ecological knowledge transmission are also scarce. This means, generally, that studies of women’s subsistence skills and material culture are underrepresented. Similarly, we only extracted two studies on learning to harvest honey. Crittenden (2011) has recently hypothesized that eating honey may have had important implications for the evolution of modern humans, and future studies should more thoroughly explore how honey harvesting is learned. The second major gap we note is the lack of studies addressing how children learn subsistence skills from one another. Specifically, we would be interested in work addressing how same-sex children teach one another particular skills. Such occurrences are mentioned obliquely in a number of our studies—boys helping one another with bows, for example—but are not developed. Given the emphasis we are seeing on peer group learning and the prevalence of vertical transmission from same-sex parents, we wonder how extensively those two trends converge in the form of same-sex children teaching one another. In addition, Africa is overrepresented in studies on learning subsistence skills. We would welcome further studies from Asia or South America. Another important oversight is the correlation between how children learn and the degree to which they rely on foraged, farmed and/or purchased food. Unfortunately, these data were rarely available in the papers surveyed, so we did not include it. However, such data would make important contributions to understanding how forager children’s learning behaviors change in association with their dependence on foraged foods. Finally, very few studies include a narrative approach in which foragers themselves explain how they learn, which would be valuable for understanding how people see their own learning process.

The present research has important implications for broadly ranging fields. Hunter-gatherer archaeologists find it especially difficult to pinpoint the role of children in the creation of the material record. Collectively, these studies demonstrate the importance of children in producing smaller versions of “adult” material culture, and they also address the complexities of human innovation as a product of entire communities, a topic that always preoccupies archaeologists. For psychologists and anthropologists particularly interested in human evolution and life history, our findings have implications for long-running debates about innovation, learning, and the reason for extended human juvenility. Furthermore, this review can facilitate comparisons with other research on small-scale agricultural, horticultural, and pastoral societies, to determine the degree to which forager learning behaviors differ from, or are similar to, those of the more commonly studied small-scale societies. Overall, it is our hope that the growing trend in studying the learning processes of foraging children continues.

## Footnotes

1 Some use more than one method. For this reason, the total is >100%.
